# Modern Technologies Supporting Motor Rehabilitation After Stroke: A Narrative Review

**DOI:** 10.3390/jcm14228035

**Published:** 2025-11-13

**Authors:** Denis Moskiewicz, Iwona Sarzyńska-Długosz

**Affiliations:** 1 Faculty of Physiotherapy, Wroclaw University of Health and Sport Sciences, 51-612 Wroclaw, Poland; denis.moskiewicz@awf.wroc.pl; 2 Rehabilitation Unit, T. Marciniak Lower Silesian Specialist Hospital, Emergency Medicine Center, 54-049 Wroclaw, Poland; 3 2nd Department of Neurology, Institute of Psychiatry and Neurology, 02-957 Warsaw, Poland

**Keywords:** post-stroke rehabilitation, neuroplasticity, robotic rehabilitation, virtual reality, functional electrical stimulation, brain–computer interfaces, non-invasive brain stimulation, combined therapies, telerehabilitation

## Abstract

**Introduction:** Stroke remains one of the leading causes of long-term disability worldwide. Post-stroke motor recovery depends on neuroplasticity, which is stimulated by intensive, repetitive, and task-specific training. Modern technologies such as robotic rehabilitation (RR), virtual reality (VR), functional electrical stimulation (FES), brain–computer interfaces (BCIs), and non-invasive brain stimulation (NIBS) offer novel opportunities to enhance rehabilitation. They operate through sensory feedback, neuromodulation, and robotic assistance which promote neural reorganization and motor relearning. **Neurobiological Basis of Motor Recovery:** Mechanisms such as long-term potentiation, mirror neuron activation, and cerebellar modulation underpin functional reorganization after stroke. **Literature Review Methodology:** A narrative review was conducted of studies published between 2005 and 2025 using PubMed, Scopus, Web of Science, Cochrane Library, and Google Scholar. Randomized controlled trials, cohort studies, and systematic reviews assessing the efficacy of these modern technologies were analyzed. **Literature Review:** Evidence indicates that RR, VR, FES, BCIs, and NIBS improve upper and lower limb motor function and strength, and enhance activities of daily living, particularly when combined with conventional physiotherapy (CP). Furthermore, integrated rehabilitation technologies (IRT) demonstrate synergistic neuroplastic effects. **Discussion:** Modern technologies enhance therapy precision, intensity, and motivation but face challenges related to cost, standardization, and methodological heterogeneity. **Conclusions**: RR, VR, FES, BCIs, NIBS, and IRT are effective complements to CP. Early, individualized, and standardized implementation can optimize neuroplasticity and functional recovery.

## 1. Introduction

Stroke remains the second leading cause of death and the third leading cause of death and disability combined worldwide [1]. It is also the primary cause of long-term disability among adults in Europe [2]. Despite advances in prevention, acute management, and rehabilitation, the global burden of stroke continues to rise due to population aging and the persistence of risk factors. Between 1990 and 2021, stroke incidence and prevalence markedly increased, and related disability-adjusted life years (DALYs) remain among the highest of all non-communicable diseases. In Europe, projections for 2017–2047 predict a 17% decrease in stroke mortality but a 27% increase in prevalence, mainly attributable to population aging and improved survival [2]. Consequently, the demand for post-stroke rehabilitation services is expected to grow even further.

Stroke, caused by either cerebral ischemia or intracranial hemorrhage [3], results in extensive neuronal damage that can lead to a broad spectrum of deficits, such as motor impairments (reduced muscle strength and altered muscle tone—spasticity/flaccidity) [4], cognitive impairments (deficits in attention, memory, language, and orientation) [5], and emotional disturbances (post-stroke depression) [6].

In addition to pharmacological treatment and endovascular interventions in the acute phase of stroke, rehabilitation aimed at restoring lost functions is critical to improving patient outcomes [7,8]. Standard methods of comprehensive post-stroke rehabilitation include conventional physiotherapy (CP) [9], occupational therapy [10], speech therapy [11], and neuropsychology [12].

Paralysis or paresis of the limbs is a common consequence of stroke, resulting in the loss or partial inability to perform movements with the affected limb [13,14], a significant issue faced by approximately 69% of survivors [15]. Therefore, post-stroke rehabilitation has become one of the most significant medical challenges in the care of this large patient group, particularly in terms of restoring independence in activities of daily living (ADL), which requires improvement in upper limb (UL) motor function.

Neuroplasticity plays a critical role in the recovery of motor functions lost due to stroke by rebuilding damaged neuronal connections, forming the foundation of rehabilitation efficacy [16]. For neuroplasticity to occur effectively, an adequate number of repetitions of motor tasks is required, as these stimulate brain reorganization and restore UL function [17]. Indeed, experimental animal studies have shown that inducing lasting changes in neural structures requires 400–600 repetitions daily [18]. However, such intervention intensity poses a major challenge in daily clinical practice, where the average number of repetitions per therapy session is only 23–32 for the UL, clearly highlighting the limitations of currently used methods [19].

Although many therapeutic methods are employed in post-stroke rehabilitation, there is still no conclusive evidence of the superiority of one over the others. However, research has consistently demonstrated that the effectiveness of rehabilitation depends on factors such as duration of the intervention, therapy intensity, and the task-oriented nature of the exercises performed [16]. As such, promising results have been achieved with robot-assisted therapy, whose effectiveness lies primarily in enabling more repetitions in less time and improving movement precision, which can significantly influence rehabilitation outcomes [20].

In response to the challenge of achieving the high training intensity required [19], the introduction of modern non-invasive and clinically applicable technologies such as robotic rehabilitation (RR), virtual reality (VR), functional electrical stimulation (FES), brain–computer interfaces (BCIs), non-invasive brain stimulation (NIBS), and integrated rehabilitation technologies (IRT) have offered new perspectives for patients, allowing them to benefit from more precise and tailored therapeutic approaches [21].

Furthermore, the development of remote rehabilitation systems enables therapy to be delivered at a distance, which is particularly important for those with limited mobility, granting them access to rehabilitation and enhancing treatment effectiveness by removing transportation and physical limitation barriers [22].

Using modern technologies in post-stroke rehabilitation also enables therapeutic progress monitoring, allowing assessment of patient functioning in daily life. Such an approach may support the process of regaining independence by providing more accurate data, allowing therapy to be better tailored to individual patient needs, and potentially improving the effectiveness of rehabilitation [23].

Despite numerous available technologies, the scientific literature remains fragmented [24], often focusing on single modalities (e.g., only BCI or only NIBS) [25,26]. This fragmentation makes it difficult for clinicians to obtain a comprehensive understanding of the technological landscape.

Addressing this knowledge gap, the present narrative review aims to synthesize and evaluate modern technologies supporting post-stroke motor rehabilitation—RR, VR, FES, BCI, NIBS, and IRT—highlighting their mechanisms, clinical applications, and potential to optimize neuroplasticity and functional recovery.

## 2. Neurobiological Basis of Motor Recovery

Motor recovery following stroke is fundamentally driven by the intrinsic capacity of the central nervous system (CNS) for neuroplasticity [27,28], a dynamic process involving structural, synaptic, and functional reorganization that can be enhanced by rehabilitation interventions [29]. The mechanisms underlying post-stroke recovery operate at both molecular and network levels [30].

At the cellular level, learning and motor relearning depend on activity-dependent synaptic modifications, primarily long-term potentiation (LTP) and long-term depression (LTD) [31,32,33].

LTP represents a persistent increase in synaptic strength following high-frequency stimulation of afferent pathways, driven by AMPA-receptor insertion and enhanced postsynaptic responsiveness [34]. Functionally, LTP underlies the strengthening of neural pathways responsible for newly acquired or recovered movements [16].

Conversely, LTD—typically induced by low-frequency stimulation—produces a sustained reduction in synaptic efficacy [35] that refines motor skills and suppresses maladaptive hyperexcitability in the contralesional hemisphere [36]. Effective rehabilitation promotes LTP-like activity in the ipsilesional cortex while inducing LTD-like inhibition contralaterally to restore inter-hemispheric balance [37].

The mirror neuron system (MNS), located mainly in the premotor (Brodmann area 6) and posterior parietal cortices [38,39,40], activates during action execution and observation [41]. MNS-based interventions—including mirror therapy and VR-assisted action observation—engage visuomotor circuits to facilitate cortical reorganization and functional recovery, especially in patients with severe paresis [42,43]. Integrating visual and proprioceptive feedback through MNS activation promotes use-dependent plasticity in the motor cortex [44].

Traditionally associated with coordination and balance, the cerebellum is now recognized as a key modulator of neuroplasticity and functional recovery via the cerebello-thalamo-cortical loop [45]. It supports error-based learning, predicting sensory outcomes, and updating motor commands [46]. Following a stroke, disinhibition of the contralesional cerebellum may contribute to pathological inter-hemispheric imbalance [47]. Targeted modulation of cerebellar activity using NIBS has improved both motor and non-motor functions, including gait, balance, dysphagia, and aphasia [48,49,50].

NIBS techniques—repetitive transcranial magnetic stimulation (rTMS), transcranial direct current stimulation (tDCS), and Paired Associative Stimulation (PAS)—modulate cortical excitability and augment neuroplastic changes induced by intensive motor training [51,52].

rTMS employs magnetic pulses to induce electrical currents in targeted cortical tissue [53], engaging LTP- and LTD-like mechanisms [54]. High-frequency rTMS (>5 Hz) and intermittent theta-burst stimulation (iTBS) enhance excitability in the ipsilesional motor cortex (M1) [55,56], whereas low-frequency rTMS (1 Hz) and continuous TBS (cTBS) inhibit contralesional M1 overactivity [57,58]. Recent reviews confirm that combining rTMS with task-specific training yields the most significant improvement in UL function when stimulation is precisely targeted [59,60].

tDCS delivers a weak, constant current that shifts neuronal resting membrane potentials [61]. Anodal tDCS increases cortical excitability (LTP-like), typically over ipsilesional M1 [62], while cathodal tDCS decreases excitability (LTD-like) over contralesional M1. Meta-analyses highlight that anodal tDCS produces significant gains in UL motor performance and ADL after stroke [63,64,65]; cerebellar tDCS also modulates motor and non-motor outcomes [48].

PAS couples peripheral-nerve electrical stimulation with a cortical TMS pulse, exploiting spike-timing-dependent plasticity (STDP) to induce lasting LTP- or LTD-like effects in corticomotor pathways [66]. Recent studies confirm PAS as a promising tool for enhancing motor recovery, particularly when combined with cerebellar TMS [67,68], and emphasize the importance of optimizing inter-stimulus intervals (ISI) for maximal efficacy [69].

## 3. Literature Review Methodology

This work is a narrative review designed to provide an integrative, comprehensive, clinically oriented synthesis of evidence on modern technologies supporting post-stroke motor rehabilitation. The aim was not to conduct a systematic quantitative analysis but rather to summarize and critically evaluate the existing literature, highlighting clinical relevance, neurobiological mechanisms, and emerging trends.

### 3.1. Data Sources and Selection Rationale

The literature search included PubMed, Scopus, Web of Science, Cochrane Library, and Google Scholar. Priority was given to randomized controlled trials (RCTs), cohort studies, and systematic reviews on the efficacy and mechanisms of modern rehabilitation technologies. Smaller pilot studies were included when offering relevant mechanistic insights, but expert opinions and editorials were excluded.

### 3.2. Search Strategy (Query Strings)

The search combined MeSH Terms and free-text keywords related to: (1) neurological dysfunction (stroke/post-stroke rehabilitation) and (2) technological interventions.

Search strings included:

- General: (“post-stroke rehabilitation” OR “stroke motor recovery” OR “hemiplegia”) AND (“modern technologies” OR “robotics” OR “virtual reality”).
- Technology-Specific: (“post-stroke rehabilitation” OR “stroke motor recovery”) AND (“robotic rehabilitation” OR “FES” OR “BCI” OR “NIBS”).
- Filtered: (“post-stroke rehabilitation”) AND (“virtual reality”) AND (“RCT” OR “systematic review”) AND (2005:2025).

### 3.3. Selection Process and Synthesis

Titles and abstracts were screened for thematic relevance, followed by full-text evaluation of eligible studies. No formal appraisal tools (e.g., GRADE or Jadad) were applied, consistent with the narrative methodology.

**Figure 1:**
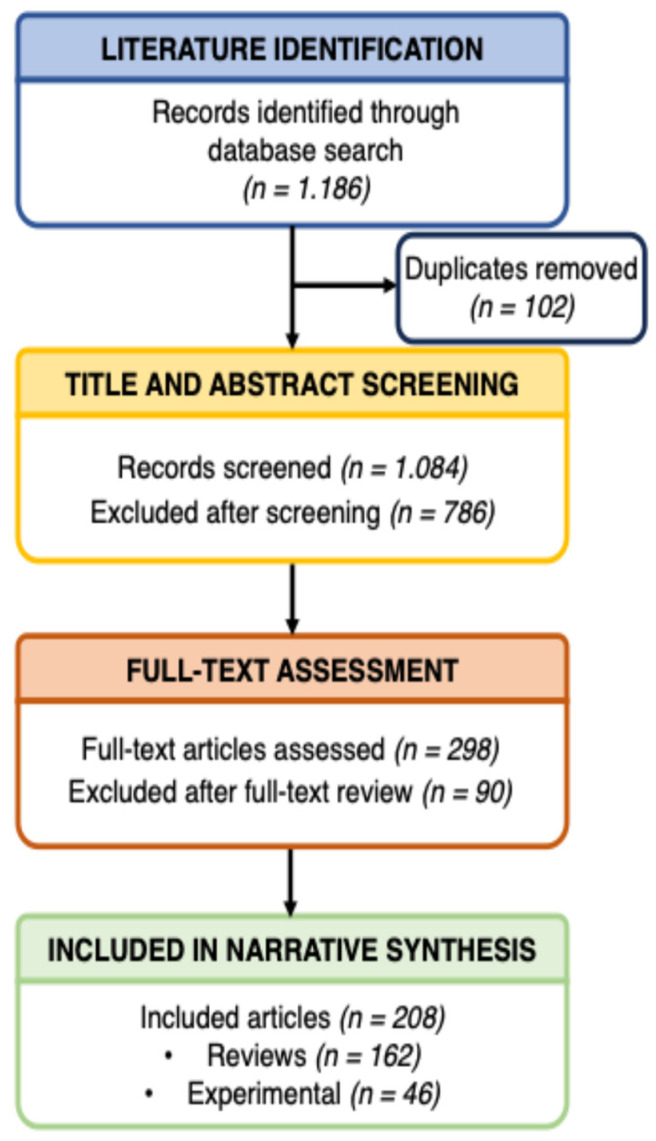
Flowchart illustrating the article selection and review synthesis process.

The search yielded 1186 records; after removing 102 duplicates, 1084 were screened. A total of 298 full-text articles were reviewed, and 90 were excluded. Ultimately, 208 studies were included: 162 reviews and 46 experimental studies. Evidence was synthesized thematically, emphasizing recurring clinical patterns, comparative efficacy, and the integration of technologies. The selection process is illustrated in Figure 1.

## 4. Literature Review

### 4.1. Robotic Rehabilitation

RR utilizes robotic devices to facilitate high-intensity, high-repetition movement training [70,71,72,73], which is fundamental for promoting neuroplasticity and cortical reorganization after stroke [74]. These robotic systems guide or assist patient movements and continuously measure joint position, force, and speed, enabling real-time adjustment of support levels and providing consistent sensory feedback that reinforces correct movement patterns [70,74]. RR supports functional recovery through precise, repetitive, and quantifiable tasks, while its application within the Task-Oriented Training (TOT) framework enhances therapeutic effectiveness [70]. This approach has become an important component of modern neurorehabilitation, offering structured, measurable, and reproducible interventions that can be integrated with CP to improve overall outcomes.

Multiple reviews confirm that RR produces small but statistically significant improvements in UL motor function, muscle strength, coordination, and ADL performance [75,76,77,78], with additional research highlighting its use in the recovery of proximal joint movements, grasping, and manipulation abilities [79]. However, the observed functional improvements are often limited and do not exceed the minimal clinically important difference (MCID), suggesting restricted clinical applicability [78,80].

Current evidence indicates that RR provides no additional benefits compared with CP when therapy dose and intensity are matched [75]. However, superior outcomes may result from the higher number of repetitions and the innovative device design, which deliver more intense neuroplastic stimulation than traditional physiotherapy [76]. Overall, these findings emphasize that RR for the UL may enhance motor performance and increase therapy intensity, but its clinical relevance depends on how effectively it is integrated into standard rehabilitation protocols.

RR, often combined with CP (e.g., Lokomat^®^—Hocoma AG, Volketswil, Switzerland, Gait Trainer—Reha-Stim Medtec GmbH, Berlin, Germany), positively affects gait pattern and postural stability [81], while electromechanical devices combined with CP increase the probability of achieving independent walking [82]. Although improvements in walking speed have been observed [83], effects on walking endurance remain limited [82].

The Lokomat^®^ system improves balance but has not shown a clear advantage over traditional physiotherapy in walking speed or functional independence [84]. Nonetheless, combining robot-assisted gait training (RAGT) with balance training and body-weight-supported treadmill training yields significant improvements in walking speed, Functional Ambulation Categories (FAC), and Berg Balance Scale (BBS) scores [83]. The positive effects are attributed to higher training intensity and the device’s ability to facilitate repetitive, task-specific movement, thereby promoting neuroplastic changes [76]. Taken together, these data suggest that RR can provide meaningful improvements in LL function and gait, particularly when applied early and combined with individualized CP programs.

The effectiveness of RR depends strongly on the timing of the intervention and the patient’s condition. RR is generally most beneficial when initiated early after stroke [82,85], with therapy started within three months maximizing UL gains [73,80]. Non-ambulatory patients benefit most in LL rehabilitation, where early intervention increases the probability of regaining independent walking [83,86]. Regardless of timing, appropriate dosing (training intensity and repetition frequency) remains a key determinant of success [72,87].

Moreover, RR allows for training intensification and a reduction in clinician workload [88]. Indeed, it optimizes resource utilization, enabling therapists to manage their time more effectively and supervise larger patient groups [70]. Despite high initial costs [70,74], long-term savings in healthcare can offset these expenses, making RR a cost-effective rehabilitation method [89].

Current evidence suggests RR is effective, particularly when therapy is tailored to the patient’s specific needs and combined with other interventions. Robotic systems offer structured and intensive training, and when integrated with CP, they may enhance functional recovery and support personalized rehabilitation strategies [85,86].

Despite these promising results, several important challenges remain. Researchers emphasize the lack of uniform guidelines regarding patient selection, optimal initiation time, and therapy dosing [73,90]. Furthermore, no superiority was found for more expensive rehabilitation devices over less costly solutions [78], which raises practical questions regarding cost-effectiveness and accessibility of RR in routine clinical practice.

The most urgent research priorities are the development of standardized guidelines for robot use [85,90], the optimization of therapeutic protocols, and the assessment of long-term effectiveness. Future research should also focus on integrating RR with VR and telemedicine technologies [74] to create more comprehensive, adaptive, and accessible rehabilitation programs. Nonetheless, RR represents a flexible and scalable rehabilitation strategy that can be adapted to different functional levels and rehabilitation phases, contributing to more efficient delivery of neurorehabilitation services.

### 4.2. Virtual Reality

VR is an advanced form of human–computer interaction that enables users to explore and interact with simulated environments replicating real-world scenarios [91,92,93]. Through coordinated visual, auditory, and occasionally tactile feedback, VR engages motor and sensory regions of the brain, enabling patients to practice functional movements in realistic environments that promote motor relearning [90,94]. In neurorehabilitation, VR is particularly valuable because it provides precise, multi-channel, real-time feedback about patient actions [94,95,96]. This feedback optimizes movement patterns and serves as the primary mechanism driving experience-dependent plasticity [16,97], which underlies motor learning [98]. VR therapy can compensate for sensory loss in stroke patients [99], thereby supporting effective motor recovery [95,97]. Importantly, VR is recognized as a safe, non-invasive, and highly adaptable therapeutic tool suitable for different stages of post-stroke rehabilitation [96].

Numerous systematic reviews and meta-analyses confirm that VR-based rehabilitation yields small but significant improvements in UL motor function [91,96,100], enhances ADL performance, and can reduce pain [91,100]. The main therapeutic advantage of VR lies in its ability to provide immediate sensory feedback and adjustable task difficulty, thereby increasing patient engagement and supporting individualized therapy goals.

Some studies suggest that VR is as effective as CP in improving UL motor function [101,102]. However, the strongest evidence supports VR as an adjunct to CP, resulting in greater, more clinically relevant improvements [103]. When combined with CP, VR helps restore muscle tone and reduce spasticity [104]. The benefits are linked to increased patient motivation and individualized task adaptation, which enhance exercise volume and intensity—critical for inducing neuroplasticity [16,97]. VR environments also promote optimal movement patterning and endpoint control through targeted sensory feedback [95]. Indeed, VR has demonstrated effectiveness in improving balance, mobility [105,106], and LL motor function. Training based on immersive environments also facilitates postural control, weight shifting, and dynamic gait components [107]. Together, these mechanisms help bridge the gap between repetitive practice and functional task transfer.

While VR enhances balance and gait, RCTs and reviews report no consistent superiority over CP in gait speed or balance outcomes [102,105,107]. Thus, although VR is beneficial, its advantage over optimized CP remains inconclusive [102]. Nevertheless, the evidence indicates that VR can complement standard gait training, especially when implemented early and combined with task-oriented interventions.

Research has evaluated VR across all stroke phases, including subacute [108,109] and chronic [101]. VR is safe, well-tolerated, and effective across diverse clinical conditions [96]. In addition, the increased engagement and exercise intensity it achieves align with the principles of TOT and neural repair [98,105].

Moreover, VR can stimulate neuroplasticity [97] and shows emerging evidence for improving cognitive functions [106]. This dual motor–cognitive impact suggests that VR may support more holistic recovery strategies that integrate physical, functional, and cognitive rehabilitation domains.

Current evidence, including systematic reviews [96,103], confirms that VR is a safe and effective method for supporting CP. Its adaptability, potential for patient engagement, and capacity to provide precise feedback make it a valuable rehabilitation tool for both UL and LL recovery. However, evidence quality remains variable [96,102,103], with common limitations including small sample sizes, heterogeneity in devices and therapeutic protocols, and short intervention durations [96,102,109]. Standardized, individualized therapy protocols are urgently needed [102,108,109]. Additionally, better patient stratification and clearer dose–response data are necessary to guide clinical implementation.

Future studies should focus on optimizing training intensity and frequency, elucidating underlying neurobiological mechanisms [97], and conducting large, multicenter RCTs to validate VR effectiveness across stroke phases [108,109] and its effects on cognition and quality of life [106]. Integration of VR with other technologies, such as RR or telemedicine platforms, may further enhance accessibility and rehabilitation efficiency.

### 4.3. Functional Electrical Stimulation

FES is a neurorehabilitation technique that uses low-energy electrical impulses to stimulate motor and sensory nerves, generating body movements and restoring motor functions such as grasping or walking [110,111]. These mild electrical signals are delivered through surface electrodes placed on the skin over the target muscles or nerves. The stimulation produces visible movements and provides the brain with sensory feedback, helping to rebuild the connection between intention and motion during recovery [112]. It is considered a promising tool for inducing neuroplastic changes and improving post-stroke motor functions. FES also allows intensive therapy delivery while reducing therapist workload and resource demands [111]. This technology can be applied across various phases of rehabilitation, supporting both UL and LL recovery through repetitive, functionally relevant training.

FES provides significant benefits in UL function, particularly when combined with TOT involving realistic activities such as grasping and object manipulation [113]. It leads to improvements in standardized test scores, including the Box & Blocks Test, the Jebsen-Taylor Hand Function Test (JTHFT), and the Fugl-Meyer Assessment for Upper Extremity (FMA-UE) [113,114]. FES also enhances ADL performance by improving grip strength and fine motor control [113,115]. A systematic review reported a moderate effect size for FES in improving physical activity and UL function [116].

Cochrane reviews indicate small but potentially significant improvements in motor function when using FES compared to no intervention [117]. The main therapeutic effect of FES is linked to increased intensity of active motor practice and improved sensorimotor feedback, supporting more efficient motor relearning.

FES enhances muscle strength, coordination, and range of motion, while helping restore voluntary contractions and reduce spasticity [116,118]. It may also decrease reliance on assistive orthoses [119]. Bilateral FES training has shown additional benefits, particularly for wrist and hand movements [114]. These effects are especially valuable during the early recovery phase, when neuroplastic potential is at its peak.

When combined with CP, FES improves LL motor functions, walking speed, cadence, and step length, as well as ankle range of motion [120,121,122]. Patients receiving FES achieve better LL motor control, gait ability, and ADL outcomes than controls [121,122]. Such improvements are clinically relevant because they translate into greater independence in mobility and daily functioning.

Cochrane reviews indicate small but potentially significant gains over no intervention; however, comparisons with placebo or traditional therapy show no consistent superiority [117,123]. As such, the overall advantage of FES over standard gait training remains inconclusive [123].

Combining FES with rehabilitation cycling enhances LL performance and overall fitness [124], and when integrated with CP, FES improves muscle strength and structure (e.g., thickness, mass, fiber type, and metabolic activity) relative to control CP and placebo [125]. These peripheral adaptations may help sustain long-term functional gains.

The effects of FES vary by stroke phase. The greatest UL and ADL improvements occur when FES is initiated early, particularly within two months of stroke onset [115]. Early intervention improves overall ADL outcomes [126], though FES benefits are more minor or insignificant in the chronic phase (≥1 year post-stroke) [115], with case reports suggesting that intensive FES can still yield gains even at this stage [118]. However, the optimal therapeutic window remains uncertain due to limited long-term and satisfaction-focused studies [126].

Beyond motor recovery, FES is recognized as a promising tool for inducing cortical reorganization and neuroplasticity [111], supporting more efficient motor relearning and adaptive network changes. Its adaptability and potential for integration with other rehabilitation methods make it a valuable component of modern post-stroke therapy.

Current evidence supports FES as a safe and effective adjunct to CP because it can enhance both UL and LL rehabilitation, particularly when applied early and integrated with task-specific training strategies. However, evidence quality remains inconsistent [115], with notable heterogeneity across devices and stimulation protocols [117], while long-term studies are lacking. Furthermore, standardized and individualized treatment protocols are needed to optimize therapy outcomes. Further research should include larger participant samples and address the optimal parameters and timing for implementing electrical stimulation [115,117]. Additionally, combining FES with emerging technologies, such as VR and RR, may further enhance functional recovery and improve accessibility to rehabilitation services [123].

### 4.4. Brain–Computer Interfaces

BCIs are neurotechnological systems that record and interpret brain activity to enable control of external devices without the need for muscle movement [127,128]. BCIs detect neural signals associated with movement intention, typically using electroencephalography (EEG). These signals are processed by algorithms that convert them into control commands for external devices, including robotic exoskeletons, FES systems, and visual feedback displays [128,129,130].

BCI systems are considered promising technology in post-stroke rehabilitation [129,130], particularly for patients with severe motor impairments [131]. Moreover, BCIs facilitate top-down activation of sensorimotor networks and can be integrated with other rehabilitation technologies to enhance therapeutic effects.

BCI-based interventions significantly improve UL motor function [129,132], most frequently measured with the FMA-UE [127,132]. When combined with standard rehabilitation, BCIs enhance motor outcomes [128,133]. In RCTs, patients undergoing BCI therapy show greater motor improvements than those receiving CP alone [134], while combining BCI with FES produces additional benefits [132]. These synergistic effects likely reflect simultaneous central activation and peripheral stimulation, reinforcing neuroplastic mechanisms.

BCIs promote both functional and structural neuroplasticity in the CNS [135,136], especially during motor imagery (MI) training, as they activate brain areas involved in motor control, facilitating cortical reorganization [137]. EEGanalyses reveal desynchronization of motor-related rhythms during MI, confirming the engagement of relevant neural circuits [51].

BCIs also show potential in LL rehabilitation [130], with reported gains in walking speed, reduced spasticity, increased range of motion, and greater muscle strength [135,138]. Improvements in locomotion and balance have also been documented [138].

RCTs demonstrate that combined BCI training yields significant motor improvements in the UL and LL compared with CP alone [139]. Indeed, stimulating the peroneal nerve during motor planning-related brain activity supports neuroplasticity in the LL [136]. This closed-loop design may amplify functional recovery by precisely coupling cortical activation with peripheral response.

BCI effectiveness depends on the stroke stage [39], with patients in the subacute phase achieving greater UL gains than those in the chronic stage [128]. Better-preserved corticospinal pathways, as indicated by clinical and neuroimaging evidence, are associated with increased responsiveness to BCI-based rehabilitation [135,136], with benefits limited in severe hemiparesis, highlighting the need for precise patient selection [140].

BCIs may reduce depressive symptoms and improve quality of life [135,141], as demonstrated by the sustained functional gains reported after BCI therapy [139]. Moreover, personalized BCI systems may increase engagement and long-term adherence to therapy [142]. These findings support the feasibility of BCI as a clinically relevant adjunct to conventional rehabilitation.

Despite encouraging results, methodological heterogeneity and small sample sizes limit the strength of the evidence for BCIs in stroke recovery [140,142]. Larger multicenter RCTs with longer follow-ups are needed to confirm clinical efficacy and evaluate long-term outcomes [138,139,142]. Future research should also address protocol standardization, patient selection criteria, and integration with other rehabilitation modalities.

### 4.5. Non-Invasive Brain Stimulation

NIBS comprises techniques that modulate cortical excitability via external electromagnetic or electrical fields, including rTMS, tDCS, theta-burst stimulation (TBS), high-definition tDCS (HD-tDCS), and cerebellar stimulation. These techniques apply gentle magnetic or electrical stimulation to the scalp to activate motor-related brain regions, strengthening neural connections that support the nervous system in relearning and restoring motor functions [143]. In stroke rehabilitation, NIBS enhances neuroplasticity, rebalances interhemispheric inhibition, and potentiates behavioral training when used as priming or concurrent stimulation [144]. Meta-analyses indicate improvements in motor function, gait, balance, somatosensory processing, spasticity, and cognition [145], though these effects are moderate and variable across studies. These findings highlight the therapeutic potential of NIBS while also underscoring the need for more standardized protocols and larger clinical trials.

Meta-analyses consistently show that rTMS enhances UL and fine motor recovery after stroke, particularly with ≥20 sessions and bilateral stimulation during the acute or subacute phases [146]. Excitatory protocols (e.g., high-frequency rTMS) applied to the ipsilesional cortex appear most effective [60,147,148]. Moreover, combining tDCS with rehabilitation produces moderate gains in arm and hand function, especially when paired with CP or VR-based training [149].

Some analyses suggest that neuroplastic changes induced by rTMS may not always translate into functional superiority over CP alone [150]. However, rTMS combined with structured rehabilitation can yield additive improvements in measures such as the FMA-UE and the Action Research Arm Test (ARAT), particularly when stimulation parameters and patient selection are optimized [151]. This synergistic effect reflects NIBS’s role as an enhancer rather than a replacement for active therapy.

NIBS modulates cortical excitability gradients, facilitates LTP-like mechanisms, and recruits motor circuits for reorganization [148]. Earlier rTMS initiation (within one month) produces greater FMA improvements than delayed use [152]. These results support the importance of early intervention and tailored protocols in maximizing treatment response [153].

Systematic reviews and meta-analyses confirm that NIBS improves gait, balance, and LL motor function. Within these, tDCS and transcutaneous sDCS (tsDCS) often yield more potent effects than rTMS; bilateral montages outperform unilateral ones [154]. Gains in LL mobility and muscle strength are modest, but effects on walking speed and endurance are less consistent [155,156].

Research indicates that combining tDCS with task-specific training (e.g., treadmill training) modestly improves walking speed versus training alone, though the evidence certainty remains low [157,158]. This suggests that NIBS may be most effective as a complementary intervention rather than a stand-alone treatment. Nonetheless, simultaneous tDCS and repetitive gait training enhance neuroplasticity more effectively than sequential protocols [159], while cerebellar stimulation may further improve gait and balance by modulating the cerebello-cortical network [154,155].

The efficacy of excitatory rTMS is most significant within the first three months post-stroke, supporting the concept of a critical ‘recovery window’. However, heterogeneity across small studies limits generalizability, warranting subgroup analyses by baseline severity and lesion characteristics [60,148].

Cognitive and mood improvements have been observed in RCTs combining tDCS with cognitive therapy [160]. Meta-analyses also report benefits for dysphagia when high-intensity rTMS protocols are applied, though optimal dosing remains under investigation [161,162]. These additional effects indicate that NIBS may support not only motor but also multisystem recovery in stroke patients.

NIBS represents a valuable adjunct to CP post-stroke, as combining it with constraint-induced movement therapy (CIMT) or VR enhances motor recovery and ADL performance [163,164]. These effects position NIBS as a promising supportive tool within comprehensive rehabilitation programs. However, variability in stimulation parameters (frequency, intensity, duration, electrode placement) and small sample sizes hinder data synthesis [143,164], while standardized protocols and consistent outcome measures are lacking. As such, multicenter trials with longer follow-ups and standardized designs are required to establish efficacy and safety [164,165]. In addition, integrating neuroimaging and electrophysiological biomarkers may enable individualized treatment optimization and improve reproducibility [166].

### 4.6. Integrated Rehabilitation Technology

Multimodal neurorehabilitation strategies combine different technologies to target multiple levels of the sensorimotor system simultaneously. IRT merges diverse therapeutic modalities into a single coordinated system that links brain activity with physical movement, enabling real-time feedback and synchronized training [167,168]. By integrating central and peripheral mechanisms, such approaches can potentiate neuroplasticity, increase therapy intensity, and broaden access to active training, even in patients with severe deficits.

#### 4.6.1. VR with RR

Integrating VR with RR merges immersive sensory feedback with high-precision, repetitive motor training. Across randomized and pilot trials, VR–RR interventions demonstrate significant improvements in UL motor control, coordination, and kinematics compared with CP, especially during the acute and subacute stages of stroke [167,168,169,170,171].

These benefits stem from enhanced neuroplasticity and bilateral activation of motor networks, as realistic visual feedback and robotic precision co-stimulate cortical and subcortical pathways involved in motor relearning [168,169,170,172,173,174]. In chronic stages, effects are more minor, suggesting a time-dependent plasticity window [171]. Despite heterogeneity, evidence supports moderate effectiveness, particularly when VR tasks are individualized and aligned with robotic goals [170,173,174,175], underscoring the importance of patient-specific training paradigms.

#### 4.6.2. VR with FES

Combining VR with FES leverages central motor drive and peripheral feedback, reinforcing sensorimotor coupling. Clinical trials and systematic reviews show that VR–FES protocols outperform FES alone, improving dexterity, coordination, and range of motion, particularly in chronic hemiplegia [176,177,178]. In addition, synchronous visual-electrical feedback enhances cortical excitability and LTP-like plasticity [176,177]. Although studies are relatively small, results are consistent, indicating improved motivation, cortical reorganization, and functional outcomes [178].

#### 4.6.3. RR with FES

Hybrid RR-FES protocols synchronize mechanical assistance with neuromuscular stimulation, maximizing motor relearning. RCTs demonstrate greater improvements in strength, precision, and gait than either modality alone, particularly in early recovery [179,180,181,182]. Meanwhile, neuroimaging reveals increased cerebral perfusion and corticospinal activation, reflecting stronger central–peripheral coupling [179,181]. However, mixed findings versus intensive physiotherapy highlight the need for standardization and patient stratification based on corticospinal integrity and residual motor capacity [180,182]. Further research should define optimal timing, patient selection, and dosing of hybrid interventions.

#### 4.6.4. BCI-Based Combined Therapies

BCI integration adds a top-down control layer by translating neural intent into motor output. When combined with RR, FES, or VR, it enables active participation, even in patients with minimal voluntary movement, inducing measurable neuroplastic changes in EEG and fMRI (functional magnetic resonance imaging) [183,184,185,186,187].

Evidence from RCTs and pilot trials confirms significant UL motor improvement and enhanced interhemispheric connectivity after BCI-based IRT, particularly in subacute and chronic stages [165,188,189,190,191]. These systems strengthen corticomuscular coherence and restore sensory–motor feedback loops [26,165,188,190,192,193]. Despite promising results, variability in algorithms, feedback types, and sample sizes limits generalizability, emphasizing the need for large standardized trials [26,183,184,185,186,187,191,192,193].

#### 4.6.5. NIBS-Based Combined Therapies

NIBS (rTMS, tDCS) is increasingly used alongside motor training, RR, or FES to prime cortical excitability and restore interhemispheric balance [194,195,196,197]. Meta-analyses reveal additive effects when excitatory ipsilesional or inhibitory contralesional protocols are combined with intensive, task-specific training, especially during the subacute phase [194,195,196,197,198,199,200].

Mechanistic studies demonstrate that NIBS facilitates LTP and network reorganization, enhancing responsiveness to concurrent peripheral input [112,164,195,197,198,199]. Despite methodological variability, NIBS maintains a favorable safety profile [143,201,202]. Optimal timing—particularly stimulation before or during training—appears critical for maximizing neuroplastic outcomes [203,204].

#### 4.6.6. Summary and Clinical Implications

Collectively, multimodal neurorehabilitation approaches exert synergistic effects on neuroplasticity and functional recovery by engaging central and peripheral mechanisms concurrently. The degree of improvement depends on the stroke phase (most pronounced in early and subacute stages) [168,175,179,197], patient status (corticospinal integrity) [180,194], and underlying mechanisms such as sensorimotor feedback, cortical priming, and interhemispheric rebalancing [165,176,195,199].

Despite methodological heterogeneity, converging evidence supports individualized, mechanism-driven interventions—particularly combinations such as VR–RR, VR–FES, and BCI–FES—as the most promising strategies for optimizing post-stroke rehabilitation outcomes. Such approaches may enable more targeted, adaptive, and effective therapy across different recovery stages.

Table 1 summarizes representative studies comparing modern rehabilitation technologies in post-stroke motor recovery.

## 5. Discussion

This narrative review confirms the growing role of modern rehabilitation technologies—such as RR, VR, FES, BCI, and IRT—as effective complements to CP. These methods enable higher therapy intensity, greater precision, and improved accessibility for post-stroke patients [70,90,102,115,130,164,195]. Despite the increasing body of supporting evidence, the routine clinical implementation of these tools remains limited due to methodological heterogeneity, insufficient standardization, and cost-related barriers [85,90,102,195].

### 5.1. Clinical Implications

Modern rehabilitation technologies have markedly expanded therapeutic options in post-stroke recovery. RR provides high-intensity, repetitive, and task-oriented movement training that enhances neuroplasticity and functional improvement in the UL and LL [73,74,90]. Its principal advantage lies in its ability to deliver thousands of precise repetitions, which facilitate cortical reorganization. The most substantial benefits are observed when RR is introduced early—within the first three months post-stroke [73,80,82,85]. Meta-analyses confirm that RR improves motor coordination, muscle strength, and independence in ADL, although these gains are often moderate in magnitude [78,90].

VR technologies have become increasingly popular due to their capacity to enhance patient engagement, motivation, and therapy personalization while also supporting telerehabilitation and home-based care [96,102,103]. Systematic reviews demonstrate that VR combined with CP significantly improves UL motor function, reduces spasticity, and enhances ADL performance [100,102,103]. Moreover, the feedback-driven nature of VR environments enables individualized progression and intensity adjustments aligned with neuroplastic principles [16,97].

FES has shown strong therapeutic potential, particularly for improving UL function, grip strength, and ADL performance when applied during the subacute phase [113,114,115,116,117]. By directly stimulating motor and sensory nerves, FES promotes voluntary activation, reduces spasticity, and facilitates cortical reorganization through enhanced sensorimotor feedback [111,116,118].

BCI technology introduces a new dimension to neurorehabilitation by translating motor intention into external feedback, thereby promoting neuroplasticity, even in patients with severe paresis [130,135,136,142]. Studies have shown that combining BCI with FES or RR yields synergistic effects, improving both functional outcomes and patient motivation [165,189,190].

IRT—such as VR with RR, VR with FES, or BCI with FES—activate multiple sensory and motor pathways simultaneously, which may intensify neuroplasticity and enhance therapy efficiency [168,172,176,188]. The effectiveness of these interventions depends primarily on early initiation, precise patient selection, appropriate dosing, and sufficient repetition [72,82,115]. In the long term, RR and VR may also contribute to healthcare savings by reducing therapist workload, accelerating recovery, and promoting greater independence [85,89,102].

### 5.2. Neurophysiological Mechanisms

**Figure 2:**
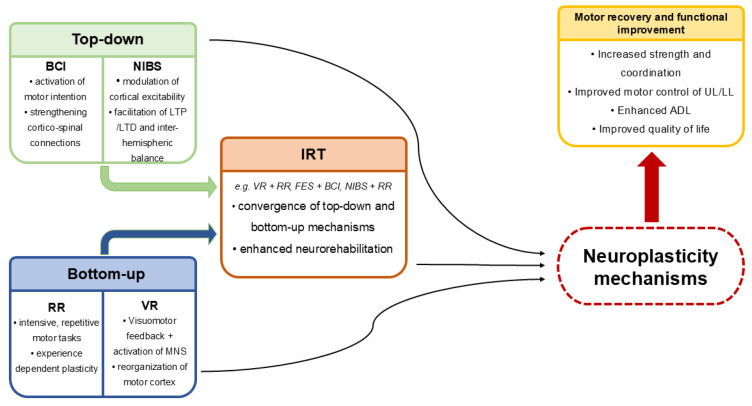
Conceptual framework illustrating the links between modern rehabilitation technologies and core neuroplasticity mechanisms in post-stroke motor recovery. BCI: Brain–Computer Interface; NIBS: Non-Invasive Brain Stimulation; LTP: Long-Term Potentiation; LTD: Long-Term Depression; RR: Robotic Rehabilitation; VR: Virtual Reality; MNS: Mirror Neuron System; IRT: Integrated Rehabilitation Technology; FES: Functional Electrical Stimulation; UL: Upper Limb; LL: Lower Limb; ADL: Activities of Daily Living.

Post-stroke motor recovery fundamentally relies on neuroplasticity—the brain’s capacity to reorganize and form new functional connections [16,27,30]. Modern technological interventions facilitate this process by providing repetitive, goal-directed, and feedback-based experiences that strengthen relevant neural pathways [16,97]. A conceptual overview of the mechanisms linking neuroplasticity to the discussed modern rehabilitation modalities is presented in Figure 2.

RR and VR stimulate sensorimotor networks through intensive task-specific training, promoting LTP-like effects and experience-dependent cortical remodeling [16,74,97]. Meanwhile, FES provides peripheral proprioceptive and tactile input, reinforcing sensorimotor integration and strengthening cortical representation of the affected limb [111,116].

BCI systems directly engage motor cortical activity by converting MI into real or virtual movement, thereby stimulating top-down mechanisms that restore corticomotor connectivity and interhemispheric balance [130,135,136,142]. When combined with peripheral feedback from FES or RR, BCI fosters synchronized activation of central and peripheral circuits, further enhancing functional recovery [165,188,190].

NIBS, including rTMS and tDCS, modulates cortical excitability and reduces maladaptive inhibition from the contralesional hemisphere [143,144,164,195]. These top-down (BCI, NIBS) and bottom-up (RR, FES) mechanisms converge on shared neurobiological foundations—enhanced synaptic plasticity, interhemispheric rebalancing, and improved sensorimotor feedback coupling—forming the scientific rationale for multimodal rehabilitation strategies [37,165,197].

### 5.3. Barriers and Patient Acceptance

Despite encouraging evidence, several barriers continue to limit the large-scale clinical adoption of modern rehabilitation technologies. Economic and infrastructural barriers include high acquisition and maintenance costs, particularly for robotics and BCI systems, which restrict their availability in smaller rehabilitation centers [89,205].

Organizational barriers involve the need for well-trained personnel, additional therapy time, and integration with existing rehabilitation workflows [70,102,205]. Methodological limitations include inconsistent therapeutic protocols, small sample sizes, and non-standardized inclusion criteria and outcome measures, all of which hinder meta-analytical synthesis and guideline development [90,102,164].

Patient-related challenges—such as cognitive impairment, mental fatigue, sensory overload (especially in VR), or discomfort during stimulation (in FES)—may reduce compliance [96,115,117]. Nevertheless, studies report generally high levels of motivation and satisfaction, particularly when devices are intuitive, adaptive, and therapist-supervised [102,174,206].

Effective clinical adoption requires intuitive interfaces, adjustable task difficulty, and real-time progress tracking to enhance engagement and adherence [97,103,130,207]. Equally important are comprehensive training programs for healthcare professionals and the establishment of safety and reimbursement frameworks to ensure responsible implementation of technology-assisted rehabilitation [205,206].

### 5.4. Future Directions

Future research should focus on consolidating current evidence and facilitating the integration of technological rehabilitation tools into standard clinical care. Indeed, large-scale, multicenter RCTs comparing single and multimodal interventions are required, with standardized protocols and long-term follow-up to assess the durability of effects [90,102,115,138,164].

Furthermore, developing adaptive and personalized systems that utilize real-time neurophysiological and biomechanical feedback (EEG, EMG [electromyography], and motion sensors) will enable more precise, responsive therapy that is tailored to patient needs [130,136,142,165].

The expansion of telerehabilitation and wearable technologies represents a key opportunity to improve accessibility and continuity of care, especially for patients with mobility limitations or those living in remote areas [22,206,208].

Integrating neuroimaging and electrophysiological biomarkers into research and clinical practice will deepen understanding of underlying mechanisms and help identify treatment responders [29,143,166]. Additionally, cost-effectiveness analyses and economic modeling are necessary to support health policy decisions and justify investments in advanced rehabilitation systems [85,89,195,205].

Ultimately, interdisciplinary collaboration among clinicians, neuroscientists, engineers, and policymakers will be essential to translate emerging technologies into effective, equitable, and sustainable rehabilitation strategies for stroke survivors [205,207].

### 5.5. Limitations

This narrative review has several limitations. The study was not preregistered, which may increase the risk of selection bias. Considerable heterogeneity among the included studies—in terms of populations, intervention parameters, and outcome measures—limits direct comparison and precludes meta-analytical synthesis. The majority of research focuses on UL rehabilitation, whereas studies on LL recovery remain underrepresented.

Furthermore, there is a lack of long-term follow-up and limited reporting of adverse effects, which restricts assessment of treatment durability and safety. Most available studies are small-sample and single-center, often lacking standardized dosing protocols, which reduces generalizability and underscores the need for methodological harmonization and international consensus in future neurorehabilitation research.

## 6. Conclusions

Modern neurorehabilitation technologies, including RR, VR, FES, BCI, and NIBS, represent a major advancement in post-stroke motor rehabilitation. These interventions enable intensive, individualized, and feedback-based training that promotes neuroplasticity and enhances functional recovery when integrated with CP, particularly in the early post-stroke phase. Their use reflects a shift from passive rehabilitation toward active, mechanism-driven interventions that engage both central and peripheral pathways.

Evidence consistently indicates that the use of modern technologies improves UL and LL motor functions, increases patient motivation, and optimizes therapy precision. However, the current body of evidence remains moderate in quality, and the clinical superiority of these interventions over CP has not been conclusively demonstrated. Key research gaps persist, including the lack of large, multicenter RCTs, the absence of standardized therapeutic protocols, and limited data on long-term outcomes and cost-effectiveness.

Based on the reviewed literature, the following conclusions can be drawn:

1. RR, VR, FES, BCI, and NIBS demonstrate measurable benefits in motor recovery, patient engagement, and individualized therapy.
2. The highest therapeutic effectiveness is achieved when these technologies are implemented early after stroke and combined with CP.
3. IRT (e.g., VR with RR, FES with BCI) produces synergistic effects by simultaneously activating central and peripheral mechanisms, thereby enhancing neuroplasticity and functional recovery.
4. Clinical application of modern technologies should be based on individualized assessment, standardized dosing and timing parameters, and clearly defined qualification criteria.
5. Future research should focus on high-quality RCTs, harmonized protocols, long-term follow-up, and the integration of neurophysiological biomarkers (EEG, EMG, fMRI) to identify optimal responders and mechanisms of recovery.

In summary, modern neurorehabilitation technologies offer a powerful opportunity to transform post-stroke motor recovery. When applied early, guided by individualized protocols, and combined into multimodal strategies, they can enhance neuroplasticity, amplify functional gains, and improve patient engagement beyond the capabilities of conventional therapy alone. With the development of standardized protocols and high-quality clinical evidence, these approaches have the potential to become an integral pillar of evidence-based stroke rehabilitation worldwide.

## Figures and Tables

**Table 1:** Comparative characteristics of modern rehabilitation technologies after stroke. RR: Robotic Rehabilitation; VR: Virtual Reality; FES: Functional Electrical Stimulation; PNS: Peripheral Nervous System; EEG: Electroencephalography; BCI: Brain–Computer Interface; NIBS: Non-Invasive Brain Stimulation; IRT: Integrated Rehabilitation Technology; ↑: increase; →: leads to.

Technology	Optimal Phase	Desired Patient Status	Mechanism	Implementation Barriers
RR	Acute–subacute (≤3 months)	Partial voluntary movement; non-ambulatory	High-intensity, repetitive, task-specific training; neuroplasticity induction	High cost, staff training, limited access, and no standardization
VR	Subacute–chronic	Cognition intact; good visual attention	Multisensory feedback, motivation ↑, experience-dependent plasticity	Device heterogeneity, sensory overload, dizziness, and poor protocol unification
FES	Early subacute (≤2 months)	Partial/absent voluntary movement; preserved PNS	Motor/sensory nerve stimulation, muscle contraction, sensorimotor coupling	Discomfort, contraindications, weak chronic-phase effects
BCI	Subacute–chronic	Severe paresis/minimal movement	Cortical activity → external feedback; top-down neuroplasticity	High cost, EEG instability, technical complexity, time-intensive
NIBS	Acute–subacute	Conscious, intact cortical integrity	Modulation of cortical excitability; interhemispheric rebalancing	Variable response, protocol inconsistency, operator-dependent
IRT	Mainly subacute	Individualized	Synergistic central–peripheral activation; neuroplasticity ↑	High cost, organizational complexity, interdisciplinary coordination

## Data Availability

No original data are presented in this narrative review.

## Abbreviations

The following abbreviations are used in this manuscript:

ADL	Activities of Daily Living
ARAT	Action Research Arm Test
BBS	Berg Balance Scale
BCI	Brain–Computer Interface
CIMT	Constraint-Induced Movement Therapy
CNS	Central Nervous System
CP	Conventional Physiotherapy
cTBS	Continuous Theta-Burst Stimulation
DALYs	Disability-Adjusted Life Years
EEG	Electroencephalography
FAC	Functional Ambulation Categories
FES	Functional Electrical Stimulation
fMRI	Functional Magnetic Resonance Imaging
FMA	Fugl-Meyer Assessment
FMA-LE	Fugl-Meyer Assessment for Lower Extremity
FMA-UE	Fugl-Meyer Assessment for Upper Extremity
HD-tDCS	High-Definition Transcranial Direct Current Stimulation
IRT	Integrated Rehabilitation Technology
ISI	Inter-Stimulus Intervals
iTBS	Intermittent Theta-Burst Stimulation
JTHFT	Jebsen-Taylor Hand Function Test
LL	Lower Limb
LTD	Long-Term Depression
LTP	Long-Term Potentiation
MCID	Minimal Clinically Important Difference
MI	Motor Imagery
MNS	Mirror Neuron System
NIBS	Non-Invasive Brain Stimulation
PAS	Paired Associative Stimulation
PNS	Peripheral Nervous System
RAGT	Combining Robot-Assisted Gait Training
RCT	Randomized Controlled Trial
RR	Robotic Rehabilitation
rTMS	Repetitive Transcranial Magnetic Stimulation
STDP	Spike-Timing-Dependent Plasticity
TBS	Theta-Burst Stimulation
tDCS	Transcranial Direct Current Stimulation
TOT	Task-Oriented Training
UL	Upper Limb
VR	Virtual Reality
